# [(1*R*,3*S*)-2,2-Dichloro-3-(hy­droxy­meth­yl)cyclo­prop­yl]methanol

**DOI:** 10.1107/S1600536813000366

**Published:** 2013-01-12

**Authors:** Mohammed H. Kailani

**Affiliations:** a Department of Chemistry, The University of Jordan, Amman 11942, Jordan

## Abstract

The title compound, C_5_H_8_Cl_2_O_2_, represents a *meso* isomer crystallizing in a chiral space group with two mol­ecules per asymmetric unit. The mol­ecules form helical associates with a pitch of 6.31 Å along the *a* axis *via* O—H⋯O hydrogen bonds. The overall three-dimesional supra­molecular architecture is stabilized by C—Cl⋯O halogen bonding, with a Cl⋯O separation of 3.139 (3) Å and a C—Cl⋯O angle of 162.5 (2)°.

## Related literature

For background on this class of compounds, see: Kean *et al.* (2012 ▶); Lenhardt *et al.* (2009 ▶). For one-handed helical chains caused by hydrogen bonds, see: Abe *et al.* (2012 ▶). For the preparation of this type of compound, see: Kailani *et al.* (2012 ▶); Pustovit *et al.* (1994 ▶).
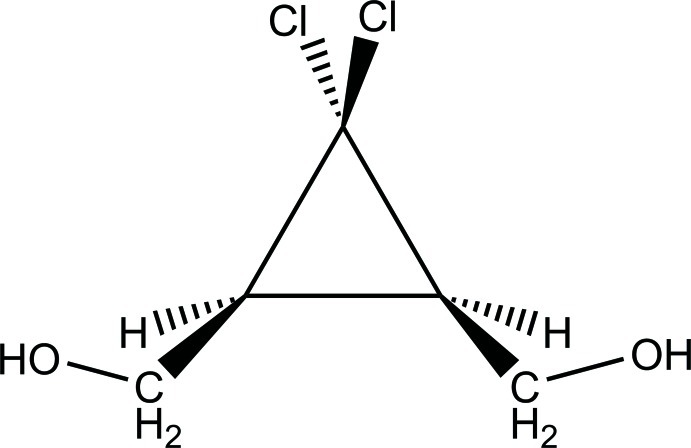


## Experimental

### 

#### Crystal data

C_5_H_8_Cl_2_O_2_
*M* *_r_* = 171.02
Orthorhombic, 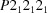

*a* = 6.3110 (13) Å
*b* = 15.429 (3) Å
*c* = 15.433 (3) Å
*V* = 1502.7 (5) Å^3^
*Z* = 8
Mo *K*α radiation
μ = 0.79 mm^−1^
*T* = 293 K
0.2 × 0.1 × 0.05 mm

#### Data collection

Nonius KappaCCD diffractometer
Absorption correction: multi-scan (*COLLECT*; Nonius, 2004 ▶) *T*_min_ = 0.91, *T*_max_ = 0.96
6911 measured reflections
2628 independent reflections
1969 reflections with *I* > 2σ(*I*)
*R*_int_ = 0.047

#### Refinement

*R*[*F* ^2^ > 2σ(*F* ^2^)] = 0.038
*wR*(*F* ^2^) = 0.085
*S* = 1.03
2627 reflections
181 parameters
H atoms treated by a mixture of independent and constrained refinement
Δρ_max_ = 0.17 e Å^−3^
Δρ_min_ = −0.17 e Å^−3^
Absolute structure: Flack (1983 ▶), 1081 Friedel pairs
Flack parameter: 0.03 (9)

### 

Data collection: *COLLECT* (Nonius, 2004 ▶); cell refinement: *SCALEPACK* (Otwinowski & Minor, 1997 ▶); data reduction: *DENZO* (Otwinowski & Minor, 1997 ▶) and *SCALEPACK*; program(s) used to solve structure: *SHELXS97* (Sheldrick, 2008 ▶); program(s) used to refine structure: *SHELXL97* (Sheldrick, 2008 ▶); molecular graphics: *Mercury* (Macrae *et al.*, 2008 ▶); software used to prepare material for publication: *SHELXL97*.

## Supplementary Material

Crystal structure: contains datablock(s) I, global. DOI: 10.1107/S1600536813000366/ld2090sup1.cif

Structure factors: contains datablock(s) I. DOI: 10.1107/S1600536813000366/ld2090Isup2.hkl

Supplementary material file. DOI: 10.1107/S1600536813000366/ld2090Isup3.cml

Additional supplementary materials:  crystallographic information; 3D view; checkCIF report

## Figures and Tables

**Table 1:** Hydrogen-bond geometry (Å, °)

*D*—H⋯*A*	*D*—H	H⋯*A*	*D*⋯*A*	*D*—H⋯*A*
O1*A*—H4⋯O2*B* ^i^	0.76 (3)	1.89 (3)	2.650 (4)	174 (4)
O2*B*—H3⋯O2*A* ^ii^	0.84 (4)	1.84 (4)	2.668 (4)	171 (4)
O2*A*—H2⋯O1*B*	0.78 (4)	1.90 (4)	2.678 (4)	174 (4)
O1*B*—H1⋯O1*A* ^iii^	0.84 (4)	1.86 (4)	2.680 (5)	167 (4)

Symmetry codes: (i) 
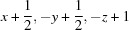
; (ii) 
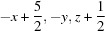
; (iii) 
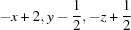
.

**Fig. 1.**
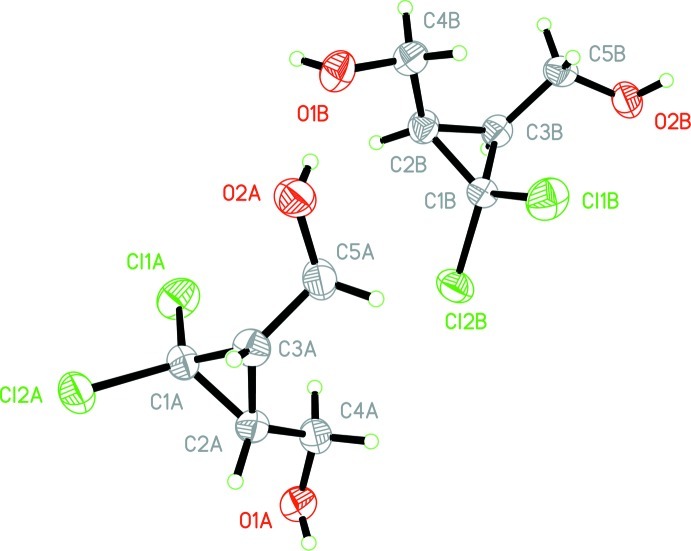
Two independent molecules in asymmetric unit with numbering scheme. Displacement ellipsoids are drawn at the 30% probability level.

**Fig. 2.**
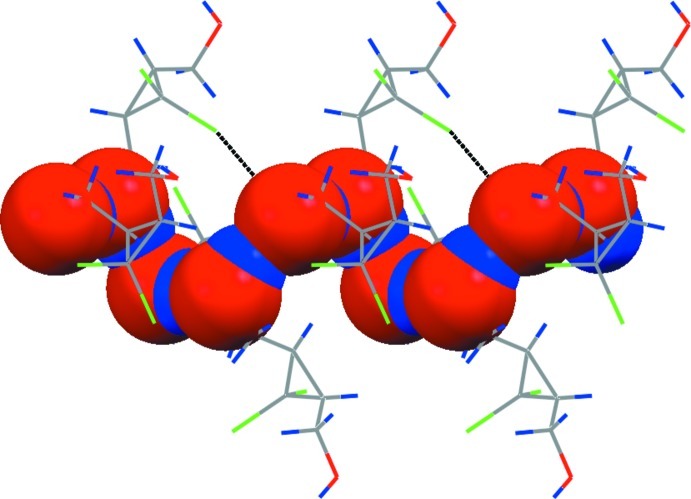
Left-handed hydrogen-bonded helix (oxygens are in red and hydrogens - in blue), running along a-axis with supporting halogen bonding interactions, shown as black dotted lines.

**Fig. 3.**
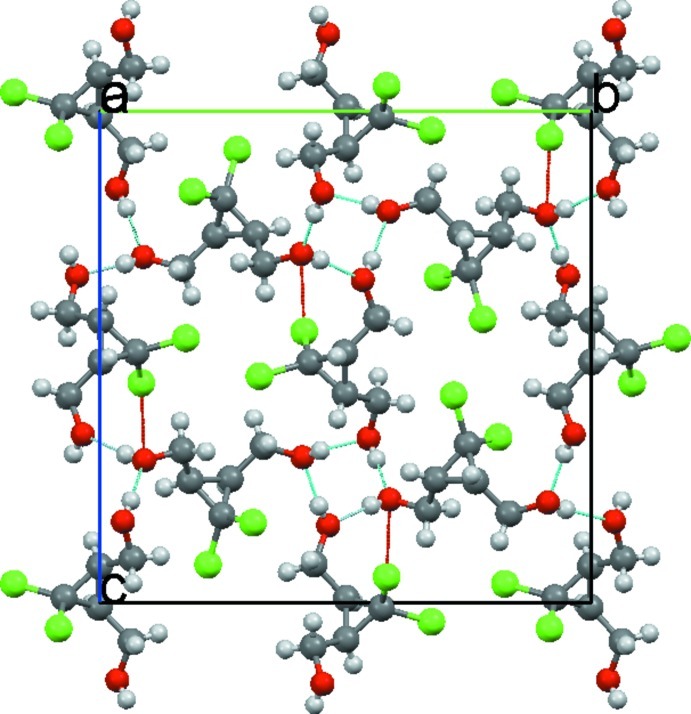
Packing diagram, viewed down the a-axis. Hydrogen bonds are shown as blue dotted lines and halogen bonds shown as red dotted lines.

## supplementary crystallographic information

### Comment

The title compound contains *gem*-dichlororcyclopropane ring with two symmetrically positioned hydroxy groups. *gem*-dichlororcyclopropane ring was recently recognized as a mechanophore by Lenhardt *et al.* (2009) and the title compound was used by Kean *et al.* (2012) to make polymers with mechanophore properties. Recently, Kailani *et al.* (2012), also reported enhancement of antimicrobial activity for novel *cis*-dicarbamates prepared from title compound.

Although the title compound is a *meso*-isomer, it crystallizes in a chiral supramolecular architecture. Abe *et al.* (2012) suggested that the presence of two hydroxy groups, which form intramolecular hydrogen bonds, can result in formation of one-handed helical structures in polymers, even in an absence of chiral moieties.

The title compound, (I), crystallizes with two molecules in the asymmetric unit, as shown in Fig. 1. Although the title compound is expected to be achiral in solution due to presence of the internal plane of symmetry, in the solid state both of the molecules are found to lack a plane of symmetry. In addition, both of said molecules were found to be not superimposable with each other, resulting in a chiral, orthorhombic P2(1)2(1)2(1) space group. The structure also has a long range chiral order, helical hydrogen bonded O—H···O chains with a pitch of 6.311 Å, running along the *a* axis (Fig. 2). These chains are further reinforced by C1B—Cl1B···O1A halogen bonding interactions with interaction parameters of 3.139 (3) Å and 162.5 (2)° resulting in the three-dimensional supramolecular structure, as shown in Fig. 3.

The lack of plane of symmetry in each molecule in the asymmetric unit is mainly caused by the differences in the spatial arrangements of the oxygen atoms within each molecule. This is thought to be caused by the presence of high concentration of strongly associating groups, two hydroxy groups and two Cl atoms, in the relatively small title compound.

### Experimental

The title compound was prepared according to literature procedure, Kailani *et al.* (2012), which was a modification of reported procedure, Pustovit *et al.* (1994), in order to improve the yield.

### Refinement

The structure represents a merohedral twin with 8.2% contribution of the opposite chirality. All carbon-attached hydrogen atoms were placed in the calculated positions using riding model with *U*_eq_. of 1.2 times that of the riding atom. Oxygen-attached hydrogen atoms were located from Fourier map difference, and then refined isotropically without restraints.

### Crystal data

**Table:**

C_5_H_8_Cl_2_O_2_	*D*_x_ = 1.512 Mg m^−^^3^
*M**_r_* = 171.02	Melting point = 346–347 K
Orthorhombic, *P*2_1_2_1_2_1_	Mo *K*α radiation, λ = 0.71073 Å
Hall symbol: P 2ac 2ab	Cell parameters from 2370 reflections
*a* = 6.3110 (13) Å	θ = 1.0–27.5°
*b* = 15.429 (3) Å	µ = 0.79 mm^−^^1^
*c* = 15.433 (3) Å	*T* = 293 K
*V* = 1502.7 (5) Å^3^	Chunk, colorless
*Z* = 8	0.2 × 0.1 × 0.05 mm
*F*(000) = 704	

### Data collection

**Table:**

Nonius KappaCCD diffractometer	2628 independent reflections
Radiation source: fine-focus sealed tube	1969 reflections with *I* > 2σ(*I*)
Graphite monochromator	*R*_int_ = 0.047
Detector resolution: 9 pixels mm^-1^	θ_max_ = 25.0°, θ_min_ = 1.3°
CCD scans	*h* = −7→6
Absorption correction: multi-scan (*COLLECT*; Nonius, 2004)	*k* = −18→18
*T*_min_ = 0.91, *T*_max_ = 0.96	*l* = −12→18
6911 measured reflections	

### Refinement

**Table:**

Refinement on *F*^2^	Hydrogen site location: inferred from neighbouring sites
Least-squares matrix: full	H atoms treated by a mixture of independent and constrained refinement
*R*[*F*^2^ > 2σ(*F*^2^)] = 0.038	*w* = 1/[σ^2^(*F*_o_^2^) + (0.0363*P*)^2^] where *P* = (*F*_o_^2^ + 2*F*_c_^2^)/3
*wR*(*F*^2^) = 0.085	(Δ/σ)_max_ < 0.001
*S* = 1.03	Δρ_max_ = 0.17 e Å^−^^3^
2627 reflections	Δρ_min_ = −0.17 e Å^−^^3^
181 parameters	Extinction correction: *SHELXL97* (Sheldrick, 2008), Fc^*^=kFc[1+0.001xFc^2^λ^3^/sin(2θ)]^-1/4^
0 restraints	Extinction coefficient: 0.0080 (12)
Primary atom site location: structure-invariant direct methods	Absolute structure: Flack (1983), 1081 Friedel pairs
Secondary atom site location: difference Fourier map	Flack parameter: 0.03 (9)

### Special details

**Table:**

Geometry. All e.s.d.'s (except the e.s.d. in the dihedral angle between two l.s. planes) are estimated using the full covariance matrix. The cell e.s.d.'s are taken into account individually in the estimation of e.s.d.'s in distances, angles and torsion angles; correlations between e.s.d.'s in cell parameters are only used when they are defined by crystal symmetry. An approximate (isotropic) treatment of cell e.s.d.'s is used for estimating e.s.d.'s involving l.s. planes.
Refinement. Refinement of *F*^2^ against ALL reflections. The weighted *R*-factor *wR* and goodness of fit *S* are based on *F*^2^, conventional *R*-factors *R* are based on *F*, with *F* set to zero for negative *F*^2^. The threshold expression of *F*^2^ > σ(*F*^2^) is used only for calculating *R*-factors(gt) *etc*. and is not relevant to the choice of reflections for refinement. *R*-factors based on *F*^2^ are statistically about twice as large as those based on *F*, and *R*- factors based on ALL data will be even larger.

### Fractional atomic coordinates and isotropic or equivalent isotropic displacement parameters (Å^2^)

**Table:**

	*x*	*y*	*z*	*U*_iso_*/*U*_eq_	
Cl2B	0.87935 (18)	0.17519 (6)	0.46132 (7)	0.0716 (4)	
Cl1B	1.21060 (18)	0.08413 (7)	0.55235 (8)	0.0763 (4)	
Cl2A	1.4218 (2)	0.27973 (6)	0.07758 (7)	0.0795 (4)	
Cl1A	1.08097 (18)	0.18830 (7)	0.16287 (8)	0.0769 (4)	
O1B	1.2516 (5)	−0.04682 (18)	0.3348 (2)	0.0665 (8)	
O1A	1.0243 (5)	0.40988 (19)	0.28993 (19)	0.0620 (8)	
O2B	0.7810 (5)	−0.03519 (18)	0.6574 (2)	0.0690 (9)	
C5B	0.8709 (7)	−0.0635 (2)	0.5790 (2)	0.0600 (11)	
H5BA	0.8091	−0.1187	0.5625	0.072*	
H5BB	1.0222	−0.0718	0.5864	0.072*	
O2A	1.5018 (5)	0.08789 (19)	0.2961 (2)	0.0699 (9)	
C5A	1.4000 (7)	0.1672 (2)	0.3187 (2)	0.0637 (11)	
H5AA	1.4496	0.1865	0.3750	0.076*	
H5AB	1.2481	0.1582	0.3223	0.076*	
C3B	0.8318 (6)	0.0018 (2)	0.5091 (2)	0.0497 (9)	
H3BA	0.6823	0.0172	0.5015	0.060*	
C4A	1.1354 (6)	0.3326 (2)	0.3103 (3)	0.0583 (11)	
H4AA	1.0382	0.2839	0.3078	0.070*	
H4AB	1.1897	0.3365	0.3689	0.070*	
C4B	1.1321 (7)	−0.0657 (2)	0.4107 (3)	0.0626 (11)	
H4BA	1.2261	−0.0667	0.4604	0.075*	
H4BB	1.0689	−0.1227	0.4051	0.075*	
C2B	0.9584 (6)	0.0010 (2)	0.4257 (3)	0.0529 (10)	
H2BB	0.8767	0.0159	0.3738	0.063*	
C3A	1.4473 (6)	0.23491 (19)	0.2521 (3)	0.0548 (10)	
H3AA	1.5983	0.2432	0.2399	0.066*	
C1A	1.3052 (6)	0.2518 (2)	0.1778 (2)	0.0507 (10)	
C2A	1.3162 (6)	0.3166 (2)	0.2490 (2)	0.0524 (9)	
H2AB	1.3973	0.3688	0.2344	0.063*	
C1B	0.9818 (6)	0.07341 (19)	0.4897 (2)	0.0486 (10)	
H4	1.095 (6)	0.448 (2)	0.302 (2)	0.047 (13)*	
H3	0.859 (6)	−0.054 (2)	0.697 (3)	0.060 (13)*	
H2	1.424 (7)	0.051 (2)	0.310 (3)	0.068 (15)*	
H1	1.181 (6)	−0.063 (2)	0.292 (3)	0.066 (14)*	

### Atomic displacement parameters (Å^2^)

**Table:**

	*U*^11^	*U*^22^	*U*^33^	*U*^12^	*U*^13^	*U*^23^
Cl2B	0.0888 (8)	0.0565 (5)	0.0694 (7)	0.0161 (6)	0.0126 (7)	0.0145 (5)
Cl1B	0.0631 (7)	0.0797 (6)	0.0862 (9)	−0.0072 (6)	−0.0204 (7)	−0.0111 (6)
Cl2A	0.1087 (10)	0.0682 (6)	0.0617 (7)	0.0140 (6)	0.0250 (8)	0.0129 (5)
Cl1A	0.0669 (7)	0.0800 (7)	0.0837 (8)	−0.0173 (6)	−0.0128 (7)	−0.0142 (6)
O1B	0.068 (2)	0.0746 (17)	0.057 (2)	−0.0007 (16)	0.0102 (19)	−0.0108 (16)
O1A	0.0628 (19)	0.0592 (18)	0.064 (2)	0.0011 (17)	0.0012 (17)	−0.0048 (14)
O2B	0.085 (2)	0.0726 (17)	0.049 (2)	0.0196 (17)	0.0000 (19)	0.0076 (15)
C5B	0.072 (3)	0.047 (2)	0.061 (3)	−0.0013 (19)	0.006 (3)	0.0043 (19)
O2A	0.078 (2)	0.0605 (18)	0.071 (2)	0.0117 (17)	0.0192 (19)	0.0079 (15)
C5A	0.073 (3)	0.070 (2)	0.048 (3)	0.016 (2)	0.005 (3)	0.0061 (19)
C3B	0.043 (2)	0.0554 (19)	0.051 (2)	0.0040 (17)	−0.0057 (19)	−0.0036 (18)
C4A	0.069 (3)	0.058 (2)	0.048 (3)	0.005 (2)	0.003 (2)	0.0028 (18)
C4B	0.073 (3)	0.057 (2)	0.058 (3)	0.007 (2)	−0.003 (3)	−0.0065 (19)
C2B	0.052 (2)	0.058 (2)	0.049 (2)	0.0065 (18)	−0.001 (2)	−0.0027 (17)
C3A	0.047 (2)	0.058 (2)	0.059 (3)	−0.0016 (19)	0.005 (2)	0.0018 (19)
C1A	0.054 (2)	0.053 (2)	0.045 (2)	−0.0014 (17)	0.003 (2)	0.0036 (18)
C2A	0.060 (2)	0.0474 (18)	0.050 (2)	−0.0061 (19)	0.004 (2)	−0.0014 (17)
C1B	0.053 (2)	0.0450 (19)	0.048 (2)	−0.0008 (17)	0.0006 (19)	0.0030 (16)

### Geometric parameters (Å, º)

**Table:**

Cl2B—C1B	1.754 (3)	C5A—H5AB	0.9700
Cl1B—C1B	1.746 (4)	C3B—C1B	1.486 (5)
Cl2A—C1A	1.767 (4)	C3B—C2B	1.515 (5)
Cl1A—C1A	1.737 (4)	C3B—H3BA	0.9800
O1B—C4B	1.423 (5)	C4A—C2A	1.502 (5)
O1B—H1	0.84 (4)	C4A—H4AA	0.9700
O1A—C4A	1.419 (4)	C4A—H4AB	0.9700
O1A—H4	0.76 (3)	C4B—C2B	1.521 (5)
O2B—C5B	1.407 (4)	C4B—H4BA	0.9700
O2B—H3	0.84 (4)	C4B—H4BB	0.9700
C5B—C3B	1.497 (5)	C2B—C1B	1.499 (5)
C5B—H5BA	0.9700	C2B—H2BB	0.9800
C5B—H5BB	0.9700	C3A—C1A	1.478 (5)
O2A—C5A	1.425 (4)	C3A—C2A	1.509 (5)
O2A—H2	0.78 (4)	C3A—H3AA	0.9800
C5A—C3A	1.496 (5)	C1A—C2A	1.487 (5)
C5A—H5AA	0.9700	C2A—H2AB	0.9800
			
C4B—O1B—H1	108 (3)	C2B—C4B—H4BB	109.3
C4A—O1A—H4	108 (3)	H4BA—C4B—H4BB	108.0
C5B—O2B—H3	107 (3)	C1B—C2B—C3B	59.1 (2)
O2B—C5B—C3B	110.2 (3)	C1B—C2B—C4B	122.2 (3)
O2B—C5B—H5BA	109.6	C3B—C2B—C4B	121.0 (3)
C3B—C5B—H5BA	109.6	C1B—C2B—H2BB	114.5
O2B—C5B—H5BB	109.6	C3B—C2B—H2BB	114.5
C3B—C5B—H5BB	109.6	C4B—C2B—H2BB	114.5
H5BA—C5B—H5BB	108.1	C1A—C3A—C5A	122.3 (3)
C5A—O2A—H2	106 (3)	C1A—C3A—C2A	59.7 (2)
O2A—C5A—C3A	110.0 (3)	C5A—C3A—C2A	119.7 (3)
O2A—C5A—H5AA	109.7	C1A—C3A—H3AA	114.7
C3A—C5A—H5AA	109.7	C5A—C3A—H3AA	114.7
O2A—C5A—H5AB	109.7	C2A—C3A—H3AA	114.7
C3A—C5A—H5AB	109.7	C3A—C1A—C2A	61.2 (2)
H5AA—C5A—H5AB	108.2	C3A—C1A—Cl1A	119.9 (3)
C1B—C3B—C5B	122.8 (3)	C2A—C1A—Cl1A	121.1 (3)
C1B—C3B—C2B	59.9 (2)	C3A—C1A—Cl2A	118.0 (3)
C5B—C3B—C2B	121.3 (3)	C2A—C1A—Cl2A	117.6 (2)
C1B—C3B—H3BA	114.1	Cl1A—C1A—Cl2A	111.1 (2)
C5B—C3B—H3BA	114.1	C1A—C2A—C4A	122.6 (3)
C2B—C3B—H3BA	114.1	C1A—C2A—C3A	59.1 (2)
O1A—C4A—C2A	112.0 (3)	C4A—C2A—C3A	122.3 (3)
O1A—C4A—H4AA	109.2	C1A—C2A—H2AB	114.0
C2A—C4A—H4AA	109.2	C4A—C2A—H2AB	114.0
O1A—C4A—H4AB	109.2	C3A—C2A—H2AB	114.0
C2A—C4A—H4AB	109.2	C3B—C1B—C2B	61.0 (2)
H4AA—C4A—H4AB	107.9	C3B—C1B—Cl1B	119.1 (3)
O1B—C4B—C2B	111.6 (3)	C2B—C1B—Cl1B	121.1 (3)
O1B—C4B—H4BA	109.3	C3B—C1B—Cl2B	118.8 (3)
C2B—C4B—H4BA	109.3	C2B—C1B—Cl2B	117.8 (3)
O1B—C4B—H4BB	109.3	Cl1B—C1B—Cl2B	111.00 (18)
			
O2B—C5B—C3B—C1B	90.9 (4)	Cl1A—C1A—C2A—C3A	109.4 (3)
O2B—C5B—C3B—C2B	163.1 (3)	Cl2A—C1A—C2A—C3A	−108.5 (3)
C5B—C3B—C2B—C1B	−112.3 (4)	O1A—C4A—C2A—C1A	−103.1 (4)
C1B—C3B—C2B—C4B	111.4 (4)	O1A—C4A—C2A—C3A	−174.7 (3)
C5B—C3B—C2B—C4B	−0.9 (5)	C5A—C3A—C2A—C1A	−112.3 (4)
O1B—C4B—C2B—C1B	−100.3 (4)	C1A—C3A—C2A—C4A	111.5 (4)
O1B—C4B—C2B—C3B	−171.0 (3)	C5A—C3A—C2A—C4A	−0.8 (6)
O2A—C5A—C3A—C1A	93.8 (4)	C5B—C3B—C1B—C2B	110.0 (4)
O2A—C5A—C3A—C2A	164.8 (3)	C5B—C3B—C1B—Cl1B	−1.6 (5)
C5A—C3A—C1A—C2A	108.1 (4)	C2B—C3B—C1B—Cl1B	−111.6 (3)
C5A—C3A—C1A—Cl1A	−3.2 (5)	C5B—C3B—C1B—Cl2B	−142.3 (3)
C2A—C3A—C1A—Cl1A	−111.3 (3)	C2B—C3B—C1B—Cl2B	107.7 (3)
C5A—C3A—C1A—Cl2A	−144.1 (3)	C4B—C2B—C1B—C3B	−109.4 (4)
C2A—C3A—C1A—Cl2A	107.8 (3)	C3B—C2B—C1B—Cl1B	108.3 (3)
C3A—C1A—C2A—C4A	−110.8 (4)	C4B—C2B—C1B—Cl1B	−1.0 (5)
Cl1A—C1A—C2A—C4A	−1.4 (5)	C3B—C2B—C1B—Cl2B	−109.3 (3)
Cl2A—C1A—C2A—C4A	140.7 (3)	C4B—C2B—C1B—Cl2B	141.4 (3)

### Hydrogen-bond geometry (Å, º)

**Table:**

*D*—H···*A*	*D*—H	H···*A*	*D*···*A*	*D*—H···*A*
O1*A*—H4···O2*B*^i^	0.76 (3)	1.89 (3)	2.650 (4)	174 (4)
O2*B*—H3···O2*A*^ii^	0.84 (4)	1.84 (4)	2.668 (4)	171 (4)
O2*A*—H2···O1*B*	0.78 (4)	1.90 (4)	2.678 (4)	174 (4)
O1*B*—H1···O1*A*^iii^	0.84 (4)	1.86 (4)	2.680 (5)	167 (4)

Symmetry codes: (i) *x*+1/2, −*y*+1/2, −*z*+1; (ii) −*x*+5/2, −*y*, *z*+1/2; (iii) −*x*+2, *y*−1/2, −*z*+1/2.
